# Performance evaluation of indented macro synthetic polypropylene fibers in high strength self-compacting concrete (SCC)

**DOI:** 10.1038/s41598-024-71875-5

**Published:** 2024-09-06

**Authors:** Chen Yaqin, Saud Ul Haq, Shahid Iqbal, Inamullah Khan, Shah Room, Shaukat Ali Khan

**Affiliations:** 1 School of Civil Engineering and Architecture, Xi’an University of Technology, Xi’an, 710048 China; 2 Department of Civil Engineering, Sarhad University of Science and Information Technology, Peshawar, Pakistan; 3 National Institute of Transportation, National University of Sciences and Technology, Islamabad, 24080 Pakistan; 4 School of Computing and Engineering, University of West London, London, UK; 5 School of Civil and Environmental Engineering, National University of Sciences and Technology, Islamabad, Pakistan

**Keywords:** Self-compacting concrete, Mechanical properties, Fibers bridging, Bond strength, Strain hardening, Flexural toughness, Civil engineering, Materials science

## Abstract

Concrete is used worldwide as a construction material in many projects. It exhibits a brittle nature, and fibers' addition to it improves its mechanical properties. Polypropylene (PP) fibers stand out as widely employed fibers in concrete. However, conventional micro-PP fibers pose challenges due to their smooth texture, affecting bonding within concrete and their propensity to clump during mixing due to their thin and soft nature. Addressing these concerns, a novel type of PP fiber is proposed by gluing thin fibers jointly and incorporating surface indentations to enhance mechanical anchorage. This study investigates the incorporation of macro-PP fibers into high-strength concrete, examining its fresh and mechanical properties. Three different concrete strengths 40 MPa, 45 MPa, and 50 MPa, were studied with fiber content of 0–1.5% v/f. ASTM specifications were utilized to test the fresh and mechanical properties, while the RILEM specifications were adopted to test the bond of bar reinforcements in concrete. Test results indicate a decrease in workability, increased air content, and no substantial shift in fresh concrete density. Hardened concrete tests, adding macro-PP fibers, show a significant increase in splitting tensile strength, bond strength, and flexural strength with a maximum increase of 34.5%, 35%, and 100%, respectively. Concrete exhibits strain-hardening behavior with 1% and 1.5% fiber content, and the flexural toughness increases remarkably from 2.2 to 47.1. Thus, macro PP fibers can effectively improve concrete's mechanical properties and resistance against crack initiation and spread.

## Introduction

Concrete is widely used in construction globally due to its affordability and the ready availability of its raw materials. However, its major disadvantage is its low tensile and flexural strength, causing it to behave brittlely with low fracture toughness^1,2^. Short discrete fibers addition to concrete makes it ductile. Vast research has already been conducted on incorporation of different types of fibers in concrete, which reported improvements its flexural strength and toughness, tensile strength, bond strength, impact, and fatigue resistance^3–6^.

While using different fibers in concrete, it is advantageous to use self-compacting concrete (SCC) to avoid external vibrators for concrete compaction, which may affect the natural orientation of fibers in concrete. SCC is distinguished by its capacity to flow under its own weight. It boasts high resistance to segregation, effectively filling formwork without the need for external vibrations to aid in compaction^7–9^. Because of this inherited property of SCC, its use improves the construction environment by lowering noise due to no use of vibrators for compaction, improves the quality of concrete, and leads to savings in terms of labor costs. To achieve high flowability, more powder content is needed in SCC, as the course aggregates require a medium for ease of movement for compaction of concrete which is through a fine aggregate-powder matrix containing higher powder content compared to normal vibrated concrete, in addition to the high dosage requirements of superplasticizers. Relying solely on cement as the powder component proves to be economically inefficient. Therefore, different mineral fillers are used, involving silica fume, fly ash, rice husk ash, blast furnace slag, glass powder and marble powder, as well as using a smaller maximum size of aggregates to facilitate unhindered flow^9–12^. Increased powder content results in finer internal structures, which may be beneficial for bonding with fibers. SCC finds its applications in nearly 40% of precast concrete production and 2–4% of cast-in-situ concrete manufacturing in the United States^13^.

Civil engineers are greatly concerned in using high-strength concrete for specialized constructions like pre-stressed concrete structures and other structures in earthquake-prone regions^14,15^. Further, fiber-reinforced high-strength SCC is a novel material combining the benefits of SCC as high flowability and fiber addition for improved strength and ductility. Different types of fibers, most commonly natural, steel, glass, and polypropylene, have been extensively added to SCC to evaluate its mechanical properties, and significant improvements have been reported^16–19^.

Different types of natural fibers have been added to concrete and results have been reported. Improvements in mechanical properties of concrete including compressive strength, tensile strength, flexural strength, toughness and ductility have been reported by incorporating hemp, kenaf and bamboo fibers into concrete^20^. Other studies have reported improvements in compressive strength, tensile strength and flexural strength with the addition of sisal fibers, basalt fibers, coir fibers and sugarcane fibers into concrete^21–23^. However there are some problems associated with natural fibers. Plant based fibers have high water absorption and sensitivity to alkaline environment in the concrete, negatively influencing the mechanical as well as durability properties of concrete^24,25^.

Steel fibers are commonly used in concrete. While the flowability of SCC may decrease, the addition of steel fibers has been reported to significantly enhance its splitting tensile strength, flexural strength, and ductility index^3,26^. The pullout behavior of reinforcements bars in concrete and tensile strength capacities of concrete were investigated by Chun et al. using macro and micro steel fibers having different geometries, including hooked end, straight, and curved fibers, reporting improved capacities because of fibers bridging effect in concrete^27^. However, steel fibers tend to corrode, reducing the concrete's service life^28^. Corrosion of steel fibers in concrete can considerably reduce their cross-section and severely affect fiber-reinforced concrete performance^29^. In addition to corrosion susceptibility, steel fibers, because of their higher unit weight, increase the self-weight of the structure, causing economic disadvantage.

Another frequently used fiber in concrete is glass fiber. Glass fibers are reported to have harmful impact on the compressive strength of concrete; nevertheless, they enhance flexural strength with cost-effectiveness^30,31^. Additionally, it has been reported that using glass fibers in low volumes can effectively improve crack resistance in SCC^32^. Another study reported slight reductions in the workability of SCC when glass fiber content was changed from 0.3 to 1.2%, but significant improvements in the splitting tensile strength of concrete while slight decrease in flexural strength were noted^33^. However, glass fibers are highly reactive in an alkaline environment^34^ as that offered by concrete. The corrosion mechanism of glass fibers in alkaline solutions primarily involves the rupture of the glass-forming –Si–O–Si– bonds, resulting in the breakdown of the entire network^35^. Therefore, these issues can affect the short- and long-term behavior of glass fiber-reinforced concrete.

Different fibers added to concrete including natural, steel and glass fibers have significant impacts on improving the properties of concrete. However, because of different issues associated with these type of fibers^25,28,29,34,35^, alternatively, Polypropylene (PP) fibers are commonly added to concrete, improving its flexural and tensile strength^19^. Concrete Owing to the PP fibers' bridging effect, its content increase in concrete results in improved concrete tensile and flexure strength^36,37^. Because of increased capacity in the post-cracking energy with the addition of PP fibers to SCC, it's pre and post-cracking toughness is improved^38^. However, traditional PP fibers are shorter, extremely thin, and soft with a smooth surface. The smooth surface of glass fibers might hinder their adhesion to the surrounding concrete, while their softness can cause a balling effect during concrete mixing. This uneven distribution of fibers can decrease concrete performance^39^. There is a rising trend in using macro fibers in concrete due to its better performance and to solve the issues associate with micro PP fibers. Twisted macro PP fibers in high strength resulted in slight improvement in compressive strength at the tune of 2.11% however, the flexural capacity of the concrete by 32.78% as compared to the control mix^40^. The addition of high-performance PP fibers exhibits the improved tensile and flexural properties of high strength concrete^41^. A previous has study reported improvements in the mechanical as well as durability properties of concrete by incorporating twisted macro polypropylene fibers into rubberized concrete. They reported improvements in post-cracking behavior, increase in fracture energy as well as improvements in resistance against drying shrinkage and frost effect^42^. Further, enhancements in flexural strength, post-cracking performance and improvements in the ductility of the concrete have been reported by previous studies incorporating 47 mm and 60 mm long crimped macro PP fibers by up to 1% v/f into concrete^43–45^. Another study reported improvements in the drying shrinkage by incorporating 0.5% to 1% v/f of 50 mm long macro PP fibers into concrete^46^. A study while evaluation the impact of addition of macro PP fibers, 30 mm in length, into concrete on the crack geometry and water permeability of concrete reported that there is increase in crack tortuosity and surface roughness, impacting the water permeability of concrete^47^. There is improvement in the compressive characteristics of recycled aggregate concrete incorporating macro PP fibers into it. Further, the samples failed in ductile manner, maintaining their integrity^48^. Thus macro PP fibers have significant positive impacts on the mechanical as well as durability properties of concrete.

### Novelty of the research

One variant of macro polypropylene (PP) fibers is engineered by bonding microfibers together to create thicker fibers, with surface undulations designed to mitigate the limitations commonly associated with micro PP fibers. The resulting macro PP fibers are expected to resist balling effects during concrete mixing process because of their thicker structure and better mechanical anchorage because of undulated surface, analogous to steel fibers. For better bonding with the surrounding matrix. Numerous researches have been carried out to study the influence of addition of these macro PP fibers with different lengths into concrete and significant enhancements in properties of concretes have been reported. Use of higher strength as well as self-compacting concrete in construction industry are increasing day by day^13–15^. Further, utilizing these fibers in self-compacting concrete may exhibit additional advantages of compacting without the use of vibrators which may change the natural orientation of fibers because of vibrator-fibers interaction. A detailed literature on the topic has been conducted, however, to the authors’ knowledge, there has been limited literature on the study of fresh and mechanical properties of high-strength SCC incorporating macro PP indented fibers. Thus, this research study investigates the fresh concrete properties, including workability, air content, and density, and mechanical properties, including compressive strength, splitting tensile strength, bond strength, and flexural strength of macro PP fiber reinforced high strength SCC. Further, detailed investigation on the post cracking behavior of macro-PP fibers added high-strength SCC has been conducted. Thus, this study may significant impact on the utilization of this type of fiber reinforced concrete for different specialized applications.

## Materials

**Fig. 1:**
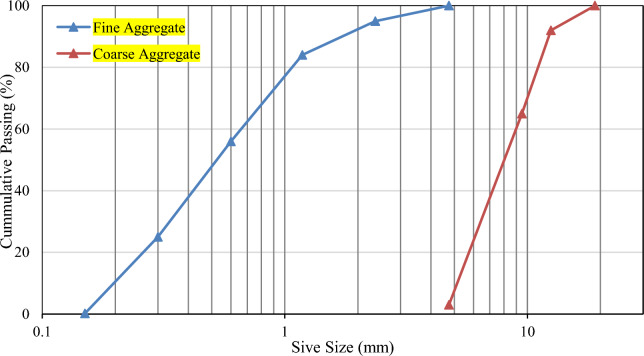
Particles size distribution of aggregates.

This research study used Ordinary Portland Cement (OPC) CEM I 42.5 R to produce concrete. 0–2 mm natural sand was employed as fine aggregate, while crushed natural aggregate had a nominal maximum aggregate size of 12 mm as a coarse aggregate particle size distribution of fine aggregate and coarse aggregate are given in Fig. 1. Class F fly ash was utilized as a filler in SCC and superplasticizer with the trade name "SIKA Viscocrete 3110", which SIKA Pakistan provided. The properties of these ingredients are summarized in Table 1.

**Table 1:** Materials properties.

Materials	Water absorption (%)	Specific gravity
Fine aggregate	1.2	2.62
Coarse aggregate	1.05	2.65
Fly ash	–	2.08
Cement	–	3.15
Superplasticizer	–	1.08

**Fig. 2:**
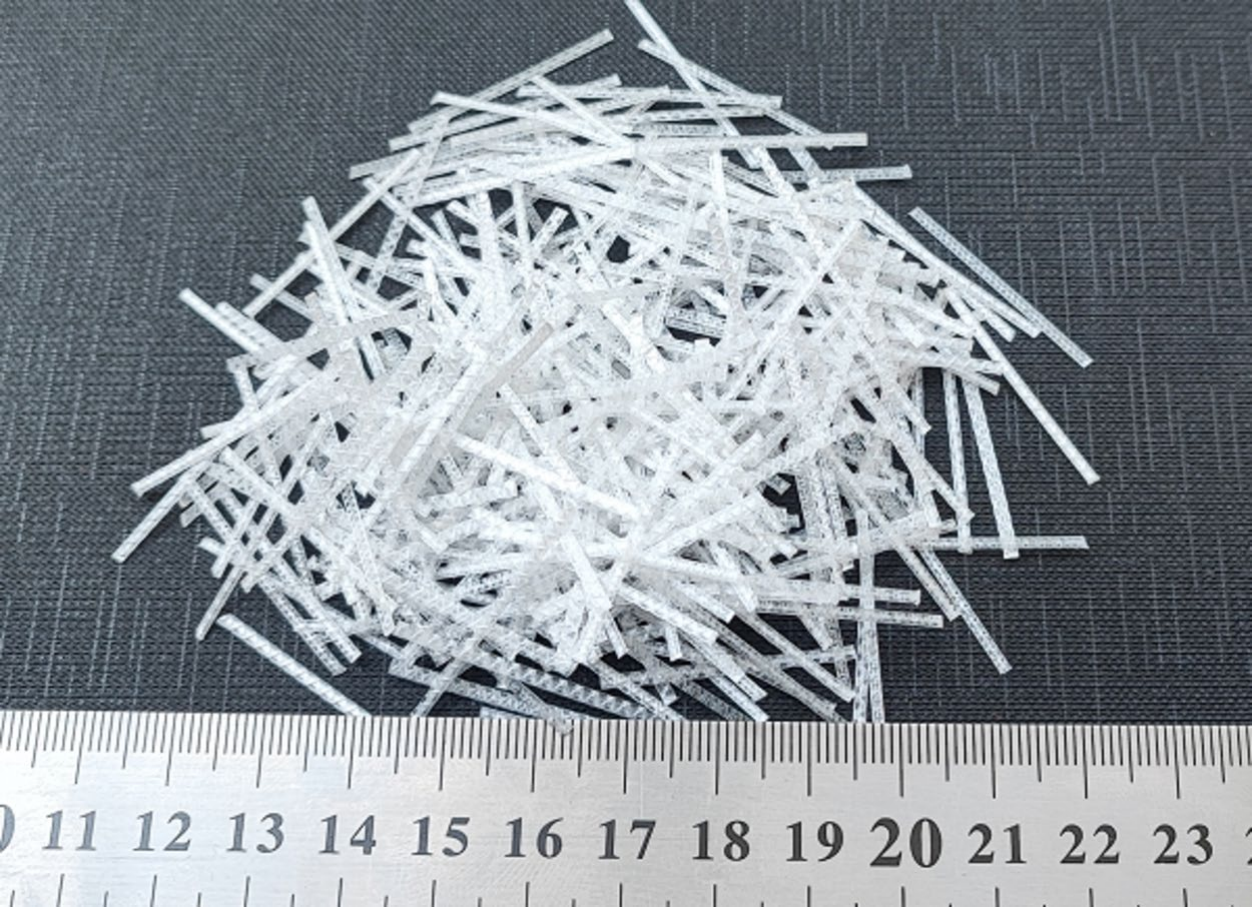
Macro polypropylene fibers.

Macro polypropylene fibers were provided by SIKA Pakistan, which are shown in Fig. 2.

The characteristics of macro PP fibers are summarized in Table 2.

**Table 2:** Characteristics of macro PP fibers.

S. no.	Details	Property
1	Color	White
2	Length	30 mm
3	Mean width	1.24 mm
4	Specific gravity	0.91
5	Melting point	160 °C
6	Ignition point	350 °C
7	Thermal and electrical conductivity	Low
8	Absorption	Nil

## Methodology

This research explored the fresh and mechanical attributes of macro PP fibers integrated into high-strength SCC. Three different concrete target strengths, 40 MPa, 45 MPa, and 50 MPa, were considered with varying fiber content of 0%, 0.5%, 1%, and 1.5%. EFNARC specifications^49^ were followed for SCC mix design development, and then trial mixes were conducted for high-strength SCC mix design finalization to be used as a control mix. Following this, fibers were introduced into the mix to analyze both the fresh and mechanical characteristics through a comprehensive experimental regimen. The concrete mixing process began with a 30-s dry mix of the coarse and fine aggregates, cement, and powder materials. Water containing superplasticizer was then gradually added, followed by an additional minute of mixing. After a one-minute rest period, the polypropylene (PP) fibers were gradually introduced, with the mixing continuing for another minute. The mixture was then ready for use. The study delved into the air content, workability, and density as fresh concrete properties. At the concrete age of 28 days, various tests including splitting tensile and compressive strength, bond strength, and flexural strength were performed in accordance with ASTM and RILEM standard testing procedures. Additionally, slump flow tests were conducted as per ASTM C1611/C1611M^50^, while ASTM C138/C138M^51^ was adopted to test the concretes' fresh concrete density and air content. The concrete samples were demolded 24 h after casting and placed in water tanks for moist curing until the testing day, following ASTM C192/C192M guidelines^52^. Three concrete cylinders had length and diameter of 200 mm and 100 mm, respectively, for compressive and splitting tensile strength of every concrete type as per ASTM C39/C39M^53^ and ASTM C496/C496M^54^, respectively. Tests for the bond strengths of reinforcements in concrete were performed as per RILEM specification^55^ because of its simplicity and wide range of applications. Three 150 mm size cube samples with 16 mm dia deformed reinforcement bars embedded into them were prepared for each type of concrete to test for the bond of reinforcements in concrete. Concrete prisms 100 mm × 100 mm × 400 mm were tested for flexural strength of each concrete as per ASTM C1609/C1609M^56^ using a four-point bending configuration. Test results are summarized and presented with detailed discussions and conclusions.

The designs of concrete mix were finalized for target strengths of 40 MPa, 45 MPa, and 50 MPa and a minimum target slump flow of 600 mm. 0.5%, 1%, and 1 0.5% v/f of macro PP fibers were included to each concrete and tests were conducted. The letter "CM" in the nomenclature represents concrete mix, the figure next to it represents the target strength, and the last figure represents the v/f of macro PP fiber. Mix ingredients of all the concretes are given in Table 3. As the specific gravity of the macro PP fibers is 0.91 or 910 kg/m^3^, the content of fibers is calculated by calculating its volume and multiplying it with its density. 0.5% v/f is calculated as under and similarly for 1% and 1.5%:

$$ {\text{v/f of macro PP content}} = {\text{Volume }} \times {\text{ Density}} $$

$$ 0.5 \% \frac{{\text{v}}}{{\text{f }}} of \, macro\, PP = \left( {\frac{0.5 \times 1\;m^{3}}{{100}}} \right) \times 910\frac{kg}{{m^{3} }} = 4.55\; kg $$

**Table 3:** Concrete mix design.

Concrete mix	Quantities (kg/m^3^)						
	PP fibers	Cement	Fly-ash	SP	CA	FA	Water
CM-40-0	0	450	100	6	800	750	198
CM-40-0.5	4.55	450	100	6	800	750	198
CM-40-1	9.1	450	100	6	800	750	198
CM-40-1.5	13.65	450	100	6	800	750	198
CM-45-0	0	450	100	6	800	750	189
CM-45-0.5	4.55	450	100	6	800	750	189
CM-45-1	9.1	450	100	6	800	750	189
CM-45-1.5	13.65	450	100	6	800	750	189
CM-50-0	0	450	100	6	800	750	180
CM-50-0.5	4.55	450	100	6	800	750	180
CM-50-1	9.1	450	100	6	800	750	180
CM-50-1.5	13.65	450	100	6	800	750	180

## Results and discussion

### Fresh concrete properties

The properties of the concrete evaluated in its fresh state encompassed workability, determined through the slump flow test, as well as air content and fresh concrete density in accordance with ASTM standard testing procedures. The outcomes of these tests are consolidated in Table 4.

**Table 4:** Fresh concrete properties.

Concrete type	Air content (%)	Workability		Density (kg/m^3^)
		Slump flow (mm)	T 500 time (s)	
CM-40-0	3.1	815	4	2415
CM-40-0.5	3.3	770	5	2400
CM-40-1	4.1	755	5	2388
CM-40-1.5	4.5	730	6	2384
CM-45-0	3.3	740	5	2421
CM-45-0.5	3.8	715	6	2392
CM-45-1	4.2	685	7	2382
CM-45-1.5	4.6	665	7	2370
CM-50-0	3.6	710	6	2410
CM-50-0.5	4.1	670	7	2385
CM-50-1	4.4	655	7	2367
CM-50-1.5	5.1	610	8	2364

**Fig. 3:**
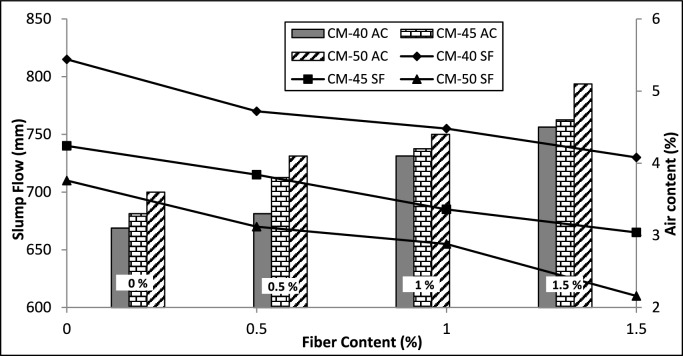
Fresh concrete properties.

The slump flow test results show a notable decrease, while the addition of macro PP fibers to concrete and an enhancement in their quantity lead to a considerable rise in air content within the concrete. Conversely, the fresh concrete density did not change considerably, which remains within a range of 2–3%. Graphical representations of air content (AC) and the slump flow (SF) results are illustrated in Fig. 3.

The maximum slump flow noted was for CM-40-0, which was 815 mm, and the minimum value was 610 mm for CM-50-1.5 but still above the target slump value of 600 mm. This reduction in slump flow is because of the hindrance the fibers provide to the concrete flow. The fibers added have slender geometry and undulations on the surface, making it difficult for the concrete to flow under its weight, reducing its workability. On the contrary, there was a substantial enhancement in the air content, which is about 40% for all concrete mixes, when the macro PP fiber contents were increased from 0 to 1.5%. This increase may be because it is difficult for the concrete to flow and compact under its weight by releasing the entrapped air. Further, because of the wrinkles on the surface of the fibers, there may be additional entrapped air in these regions, increasing the overall air content of the concrete. However, this increase in air content has no significant impact on fresh concrete density as it makes up only a few percent of the overall concrete and impacts the density slightly. The preceding study observed analogous impacts on the fresh characteristics of SCC by adding steel fibers, resulting in a marked elevation in air content and minimal alteration in density. Nonetheless, they noted a more pronounced decline in slump flow compared to the findings of the present study^3^. The comparatively lower impact on the slump flow of SCC may be ascribed to the smoother surface of macro PP fibers providing lower resistance to the flow of SCC compared to steel fibers. A study reported that the fresh properties of the SCC concrete are less effected by the use of macro PP fibers as compared to SF and polyamide fibers^57^. Incorporating up to 0.3% of micro PP fibers by weight of cement in SCC decreases its slump flow by up to 59 mm compared to the reference concrete^19^. Similar findings have been presented elsewhere^58^. In the current study, the slump flow is reduced by up to 100 mm. The greater reduction compared to micro PP fibers may be because their greater length and surface undulations hinder the concrete flow.

### Hardened concrete properties

This study comprehensively investigated the hardened concrete properties, such as compressive strength, splitting tensile strength, bond strength, and flexural strength, at 28 days of age. Each of these properties underwent comprehensive analysis and discussion.

#### Compressive strength

**Fig. 4:**
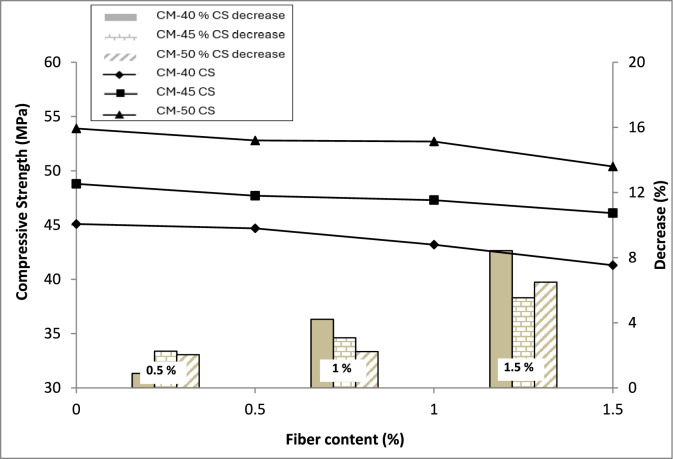
Compressive strength test results.

The compressive strength findings are presented in Fig. 4. ASTM C39/C39M standard testing practices were followed for testing the compressive strength of concrete, applying a constant load-controlled loading at 0.5 MPa/s. Test results indicate a small decrease in the compressive strength of concrete with the inclusion of macro PP fiber and an increase in its quantity.

Although the variation is insignificant, the maximum reduction in compressive strength is only 8%, exhibited by CM-40 concrete at 1.5% PP fiber content. The probable reason for this decrease may be the enhancement in the air content of concrete because of entrapped air around the fibers' geometry, which decreases the compaction of concrete and reduces its compressive strength. Further, an improvement in air content lowers the bonding between the constituents of the concrete, indicating a slight reduction in compressive strength. A previous study reported increased concrete compressive strength by incorporating micro PP fibers into SCC^19^. Similarly, another study reported an minimal increase of 2.11% in compressive strength as compared with control mix when twisted macro PP fibers having 38 mm length was used in normal high strength concrete^40^. The contradicting behavior may be attributed to the comparatively lower compatibility of macro PP fiber-reinforced SCC because of increased resistance to concrete flow and compaction. Incorporating steel fibers into SCC up to 1.25% v/f reported results similar to the current study with up to 12% reduction in compressive strength^3^, indicating that macro PP fibers behave similarly to the steel fibers.

#### Splitting tensile strength

**Fig. 5:**
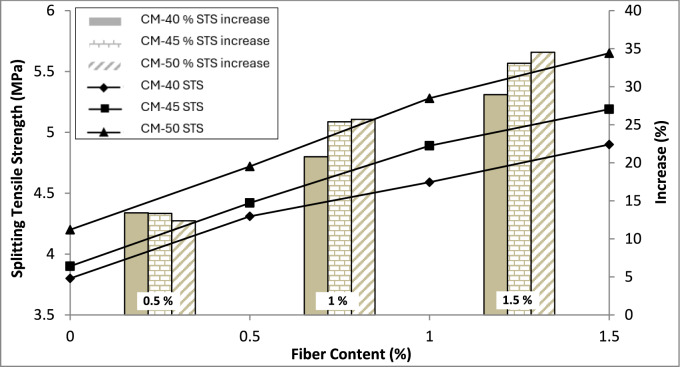
Splitting tensile strength test results.

The splitting tensile strength (STS) of the concrete was evaluated using the standard testing method specified in ASTM C496/C496M, employing a consistent loading rate of 1 MP/min. Figure 5 outlines these findings. Contrary to the compressive strength results, there is a noteworthy enhancement in concrete STS observed with the addition of fibers.

There was an increase in STS of 29–34.5% in different grades of concrete when 1.5% of PP fibers were added to them as compared to the control mix. The maximum enhancement in the STS was noted for CM-50, with an increase of 34.5% when 1.5% fibers were included compared to the control mix containing no fibers. A study reported that the use of 0.5% high performance macro PP fibers with indentations improve s the tensile strength of concrete however the strength was enhanced more when it was combined with 1% steel and 2% PP fibers^41^. A previous study has reported similar results with up to 29% increase in tensile strength of SCC strengthened with micro PP fibers^19^. Fibers are known for their bridging effect, which increases the capability of concrete against crack initiation and propagation. Increased resistance against crack initiation is why concretes' STS increased. Although once the cracks are initiated, fibers bridge them and resist the propagation of cracks, increasing the load further, this additional capacity is not counted towards the tensile strength of concrete as it has already cracked. Therefore, during this test, careful observation was made to observe the samples closely and note the loading value just when the crack was initiated in the sample.

#### Bond strength

**Fig. 6:**
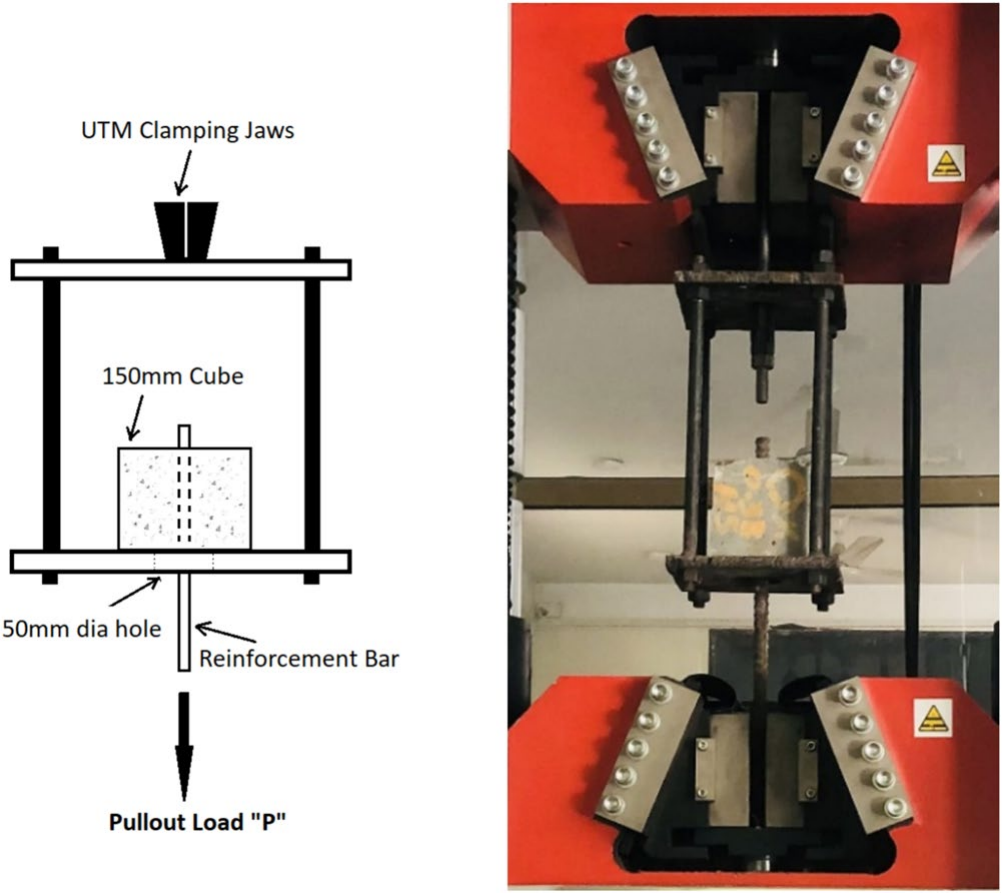
Bond strength testing arrangement.

The testing arrangement for bond strength of reinforcement bars in concrete is presented in Fig. 6.

**Fig. 7:**
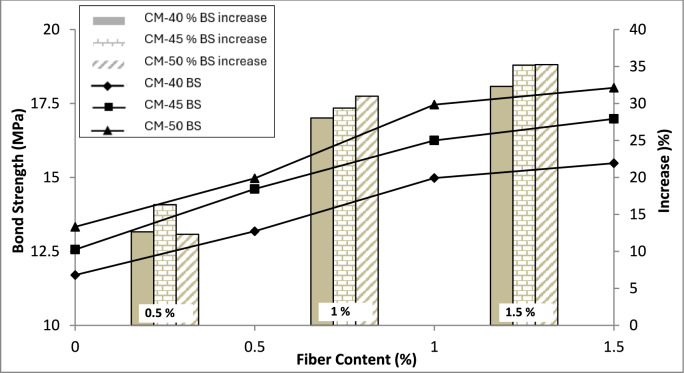
Test results of bond strength.

The test results are presented in Fig. 7. The test findings reveal that the behavior is similar to that of the splitting tensile strength.

**Fig. 8:**
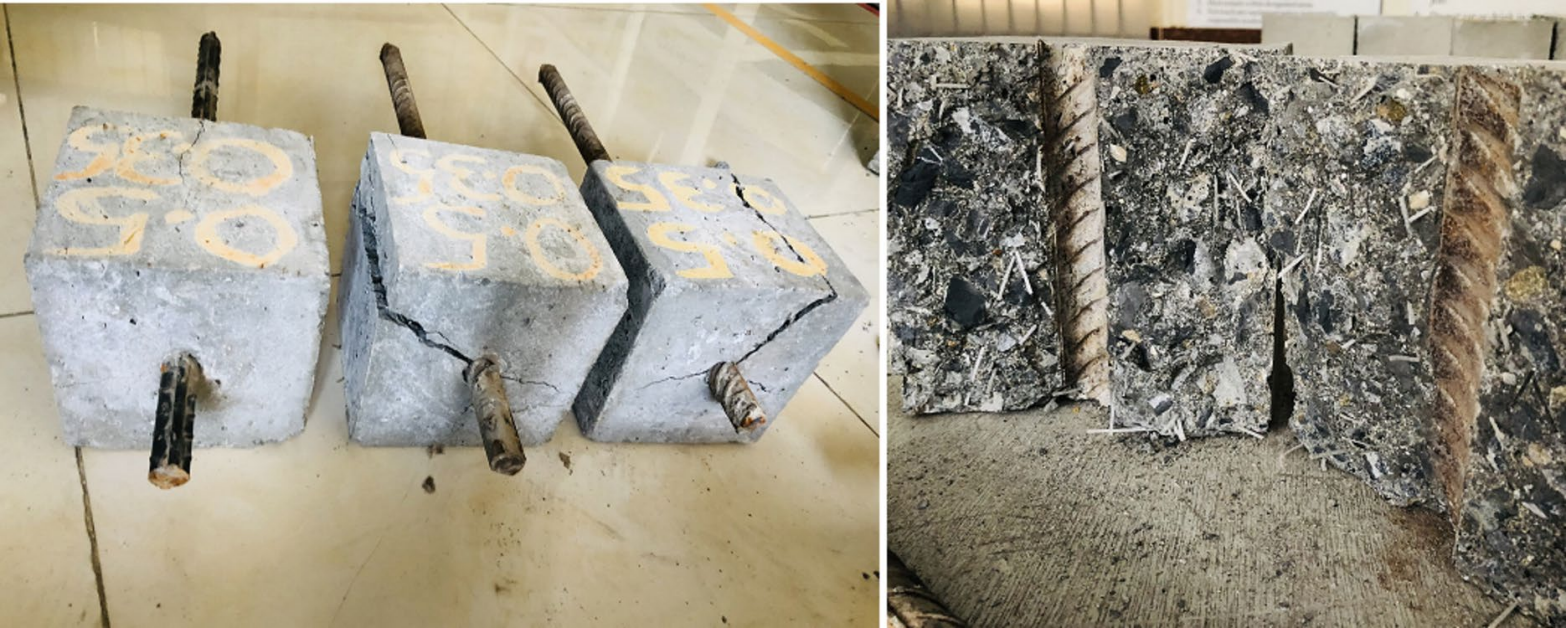
Bond strength tested samples.

The bond strength of reinforcements embedded in concrete exhibited a substantial increase of 32–35% when macro PP fibers were introduced, with the quantity escalating from 0 to 1.5% v/f. However, a slightly different trend in bond strength increase was observed. When the fibers were added up to 1% v/f, a rapid improvement in the bond strength, which is 28–31% compared to the control mix. Moreover, when the macro PP fiber quantity was further increased to 1.5%, there was only a marginal enhancement of 2–3% in bond strength values. The reason may be that fibers have achieved their maximum capacity, and further increase affects the bonding of fibers in concrete because of the increased number and increase of entrapped air around the fibers. By increasing the fiber quantity, its effectiveness increases until the optimum value is reached. As fiber content increases, the air content may be entrapped around the fibers' geometry. Additionally, because of increased fiber content, its uniform distribution may be affected because of more number of fibers interacting with each other and hindering their dispersion. This phenomena affect the bonding of fibers with the surrounding concrete as there is not enough concrete matrix for affective bonding with every fiber, resulting in reduced fiber efficiency. The bonding of fibers with concrete results in improved confinement provided by the concrete to the pullout of the embedded reinforcement bars. Once the crack is initiated because of bar pullout, the fibers resists the propagation of cracks, maintaining the confinement and resisting bar’s pullout. Reduced confinement resulting from reduced fiber bonding with the surrounding matrix leads to decline in its capacity to oppose the crack propagation and, thus, the bond of reinforcements in concrete achieves a maximum limit. Previous studies on bond strength investigation have observed similar findings with a 20 to 50% improvement in bond strength by incorporating straight and hooked-end steel fibers in SCC and normal vibrated concrete^59–61^. Another study reported improvement in bond strength up to 18% by adding deformed macro PP fibers in concrete at the tune^6^ of 4 and 6 kg/m^3^. Further, the test findings demonstrated that all the samples failed in a splitting manner, as shown in Fig. 8.

As evident from the literature, the confinement around the reinforcing bars plays an essential role in its bond strength when embedded in concrete. Because of weak aggregates, lightweight aggregate fiber reinforced concrete exhibits pullout without splitting of samples because the matrix around the bar fails and causes pullout, whereas when using normal-weight aggregate concrete, splitting failure has been reported using steel fibers^49–61^. Similar phenomena of increase in confinement occur when PP fibers are added to concrete. When a pullout load is applied to the bar, the surrounding concrete confines it, and because of mechanical anchorage due to ribs on the bar, the load is resisted. A point is reached when concrete can no longer resist these tensile stresses and cracks, initiating the sample splitting and pulling the bar out. However, in the event of PP fiber-reinforced concrete, tensile stresses are resisted because of fiber inclusion, which delays the initiation of cracks. Even when the cracks are initiated, they are not enough for the bar to be pulled out. Fibers bridge these cracks, resist their propagation, and maintain the confinement around the bar during the pullout load application. A point is reached when the bonding of fibers with the concrete fails, and they are pulled out, increasing the crack width and causing the splitting of concrete samples, creating enough area for the bar to be pulled out.

#### Flexural strength

**Fig. 9:**
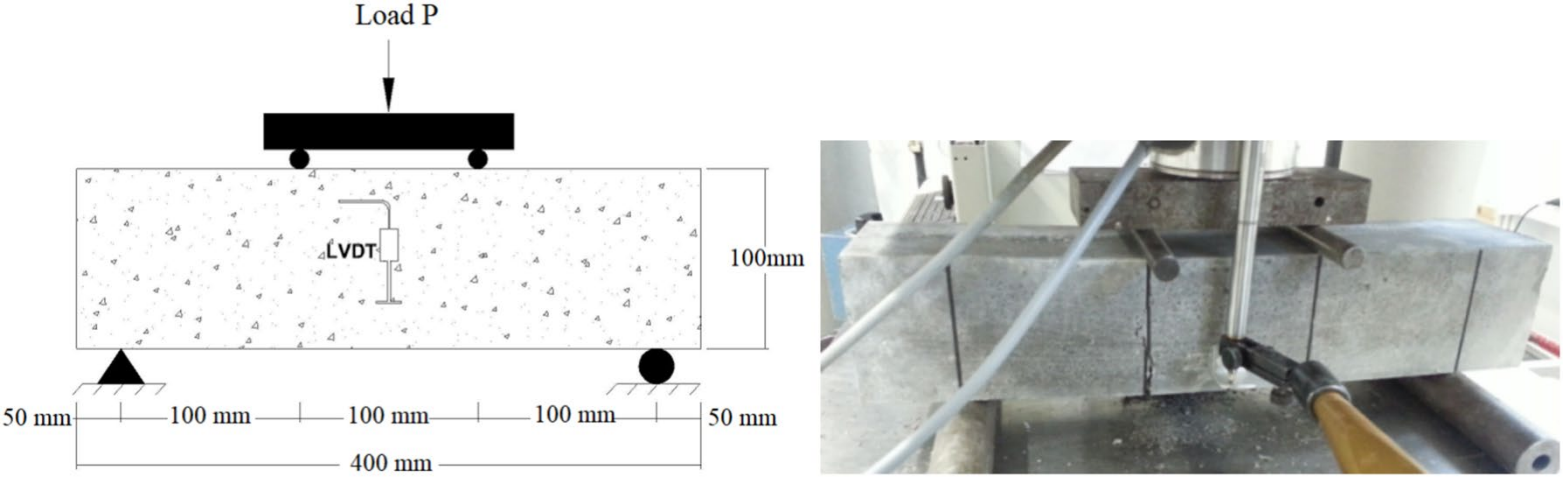
Flexural strength testing arrangement.

Flexure strength tests were conducted per the ASTM C1609/C1609M using four-point bending tests. Concrete prism samples measured cross-sectional dimensions of 100 mm × 100 mm and 400 mm in length were cast and subjected to testing with a simple span of 300 mm. LVDTs (Linear Variable Differential Transformers) were utilized at mid-span positions on both sides of the samples to record mid-span deflections. The testing setup is illustrated in Fig. 9.

**Fig. 10:**
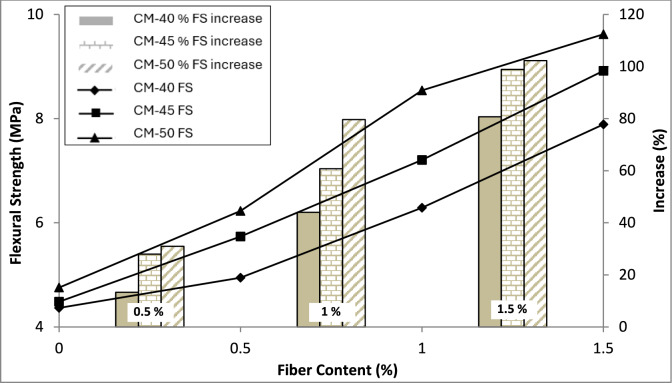
Test results of flexural strength.

The test findings, depicted graphically in Fig. 10, demonstrate a significant surge in the flexural strength of concrete upon the inclusion and escalation of macro polypropylene (PP) fibers. A remarkable 80–100% increase in concrete flexural strength is observed as the PP fiber content increased from 0 to 1.5%.

Similar to the bond strength, the rationale behind this significant improvement in flexural strength can be ascribed to the fibers bridging effect. In the case of plain concrete, the sample fails in a brittle manner when cracks are initiated in the sample; however, in the case of fiber-reinforced concrete, fibers play a vital role in resisting crack initiation. Further, when cracks occur, fibers bridge them and resist further propagation, limiting the crack width and increasing the load capacity. A point is reached when the maximum capacity of the fibers is exhausted, and they are pulled out, leading to an enhancement in the crack width, causing sample failure in a ductile fashion. The addition of micro PP fibers has observed an improvement of up to 19% in the flexural strength of SCC^19^, while another study reported a 63% increase by incorporating 0.3% micro PP fibers by weight of cement^62^. There is a 10.7% increase in the flexure strength of lightweight SCC incorporating micro PP fibers^63^. Using PP fibers in different percentages from 0 to 2% v/f, it has been reported that the flexure strength increases by up to 28% when using 1% v/f of fibers and then starts to decline, probably because of the weakened bond between components of concrete because of fibers' agglomeration^64^. A study reported increase in the flexural strength capacity of the normal M40 concrete by 32.78% as compared to the control mix^40^. However, the current study exhibited significantly higher flexure strength and increased beyond 1% v/f of PP fibers. The flexural strength of concrete increases by up to 110% when 1.25 v/f of steel fibers are incorporated into high-strength SCC^3^, which is more consistent with the current study results.

**Fig. 11:**
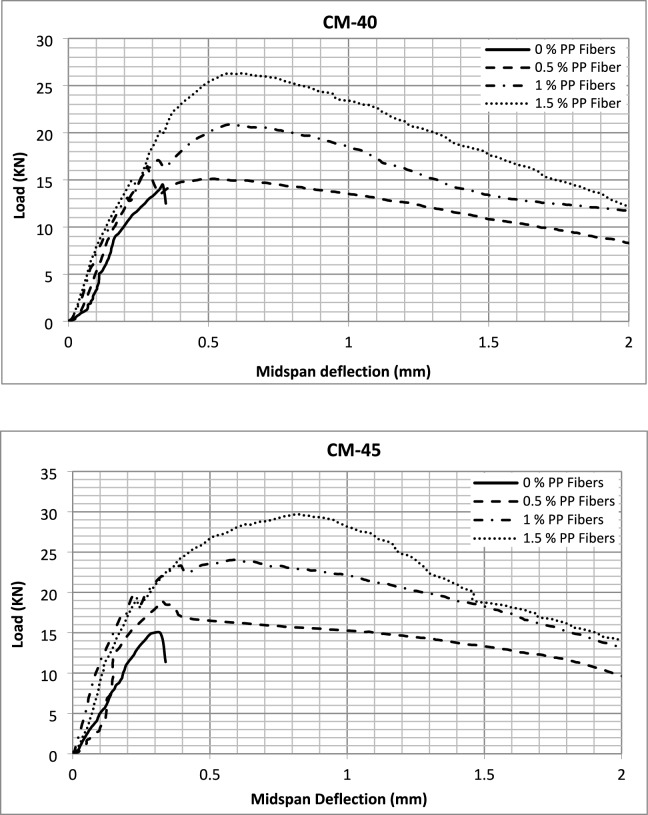

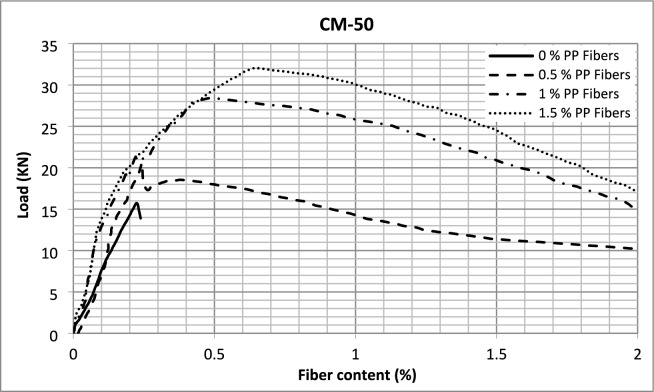
Load deflection curves.

The load–deflection curves recorded during the flexural strength tests are presented in Fig. 11. A significant finding observed from these test results was that the samples exhibited three failure mechanisms. In the case of plain concrete, the samples demonstrated brittle failure; once 0.5% fibers were included to the concrete, the failure mechanism modified from brittle to strain-softening behavior, with a significant increase in the area under the curve. However, no notable enhancement in load after first crack was observed. When the fiber quantity was further enhanced to 1% and 1.5%, the failure mechanism changed to straining hardening behavior with a further increase in load-carrying capacities after the initiation of cracks. Similar behavior is exhibited while using steel fibers in high-strength SCC^3^. However, while using different amounts of micro polypropylene fibers in lightweight SCC, it has been reported that only strain-softening behavior was exhibited by PP fiber-reinforced concrete with no further enhancement in load after the onset of first peak^65^.

The load–deflection data was further analyzed in light of ASTM C1609/C1609M to calculate vital data and information necessary for these behaviors. The calculated data is summarized in Tables 5 and 6.

**Table 5:** Load and deflection data.

Concrete type	First peak values			Ultimate values			At net displacement L/600 (0.5 mm)		At net displacement L/150 (2 mm)	
	Load (N)	Deflection (mm)	Strength (MPa)	Load (N)	Deflection (mm)	Flexural strength (MPa)	Residual load (N)	Residual strength (MPa)	Residual load (N)	Residual strength (MPa)
CM-40-0	14.52	0.336	4.36	14.52	0.336	4.36	–	–	–	–
CM-40-0.5	16.45	0.282	4.94	16.45	0.282	4.94	6.95	2.09	8.3	2.49
CM-40-1	17.1	0.320	5.13	20.92	0.576	6.29	20.03	6.01	11.73	3.52
CM-40-1.5	14.97	0.227	4.49	26.32	0.628	7.89	25.4	7.62	12.15	3.65
CM-45-0	14.94	0.320	4.48	14.94	0.320	4.48	–	–	–	–
CM-45-0.5	19.12	0.325	5.74	19.12	0.325	5.74	16.5	4.95	9.63	2.89
CM-45-1	19.96	0.224	5.99	24.04	0.587	7.21	23.58	7.07	13.08	3.92
CM-45-1.5	19.01	0.230	5.70	29.72	0.815	8.92	26.7	8.01	14.08	4.22
CM-50-0	15.85	0.228	4.76	15.85	0.228	4.76	–	–	–	–
CM-50-0.5	20.76	0.245	6.23	20.76	0.245	6.23	17.8	5.34	10.2	3.06
CM-50-1	19.92	0.187	5.98	28.45	0.490	8.54	28.41	8.52	15.08	4.52
CM-50-1.5	–	–	–	32.06	0.653	9.62	29.45	8.84	17.12	5.14

**Table 6:** Toughness and equivalent flexural strength ratio.

Concrete type	Start point (mm)	Endpoint (mm)	Toughness (J)	Equivalent FS ratio (%)
CM-40-0	0	0.348	2.4	8.3
CM-40-0.5	0	2	23.5	71.4
CM-40-1	0	2	30.8	90.1
CM-40-1.5	0	2	36.2	120.9
CM-45-0	0	0.339	2.4	8
CM-45-0.5	0	2	27.4	71.6
CM-45-1	0	2	35.5	88.9
CM-45-1.5	0	2	41.3	108.6
CM-50-0	0	0.238	2.2	6.9
CM-50-0.5	0	2	29.6	71.3
CM-50-1	0	2	41.7	104.6
CM-50-1.5	0	2	47.1	–

It can be observed from the data that the first peak values of loads in the case of plain concrete and concrete containing 0.5% fibers are the same as those of ultimate values, but there is increased flexural strength of 13%, 28%, and 30% for CM-40, CM-45, and CM-50 respectively. Flexural toughness is a measure of energy absorption capacity of concrete and gives an indication of ductility of concrete represented by the area under the load–deflection curve of the flexural strength test. Thus, the flexural toughness was calculated as area under the flexural strength load–deflection curves for further comparison. Although there is an increase in the flexural strength of concrete by incorporating 0.5% PP fibers, the major difference in their behavior is observed from their ultimate deflections and, thus, their flexural toughness values. The tests were conducted up to ultimate deflection values of L/150, which comes out to be 2 mm. There is a substantial enhancement in flexural toughness of all the concrete types incorporating macro PP fibers. This increase in toughness is because of the bridging across cracks provided by fibers, which enables the concrete samples to take load even when cracks are initiated, changing the brittle concrete failure mode to ductile. Significant residual loads and deflections exist for macro PP fiber-reinforced SCC, and the values increase with fiber content increase. Further, there is a noteworthy increase in the equivalent flexural strength ratio, indicating concrete ductility, which is the ratio of concrete's flexural toughness to first peak strength calculated using Eq. (1)

1 $$ {R_{T, 150}}^{D} = \frac{{150 \cdot {T_{150}}^{D} }}{{f_{1} \cdot b \cdot {\text{d}}^{2} }} \cdot 100 \% $$

where: ${R_{T, 150}}^{D}$ = the equivalent flexural strength ratio, ${T_{150}}^{D}$ = the flexural toughness, ${f}_{1}$ = the first peak strength, $b$ = the width of the specimen, $d$ = the depth of the specimen.

Thus, the performance of SCC is significantly improved with the incorporation of macro PP fibers into it, and the behavior shifts from brittle to ductile. Previous studies have also reported significant improvements in flexural toughness index from 0 to 19.76, adding macro PP fiber into lightweight SCC and 1 to 23 while using up to 1.25% v/f of steel fibers in high-strength SCC^3,47^. In the current study, the flexural toughness increased even more significantly than that for steel fibers, increasing from 2.2 to 47.1 at 1.5% fiber content and exhibiting strain-hardening behavior.

## Conclusions

This study investigated the influence of macro polypropylene (PP) fibers on both the fresh and mechanical properties of high-strength self-compacting concrete (SCC). Twelve distinct concrete mixes were scrutinized, each featuring varied grades and fiber contents. Drawing from this experimental investigation, we can conclude that:

1. The inclusion of macro PP fibers in high-strength SCC leads to a notable rise in air content and a reduction in slump flow. This trend becomes more evident as the quantity of PP fibers is enhanced. Conversely, there is minimal change observed in the fresh concrete density. The likely cause of the augmented air content and diminished workability can be ascribed to the impedance introduced by the added fibers to the flow and compaction of concrete, along with their propensity to trap air due to their undulated geometry.
2. The compressive strength of high-strength SCC with macro PP fibers exhibits a minor decrease, which was up to 8% with the incorporation of 1.5% v/f of fibers. The probable reason may be the non-homogeneous concrete ingredients and weaker bond between fibers and cementitious matrix compared to that between aggregates and the matrix.
3. The addition of macro PP fibers into high-strength SCC substantially improves its splitting tensile strength. This study noted up to 34.5% increase when 1.5% v/f of fibers were included into concrete. This increase may be attributed to the resistance of PP fibers to the onset of cracks in concrete by bonding the concrete ingredients together.
4. The bond strength of steel bars in high-strength SCC increases significantly with the addition of macro PP fibers, which was 35% in this study when 1.5% fibers were added. The increase in bond strength can be accredited to the enhanced confinement offered by the fiber-reinforced concrete because of the increased tensile strength capacity resisting the pullout of reinforcement bars.
5. Incorporation of up to 1.5% v/f of macro PP fibers into high-strength SCC increases its flexural strength by a significant amount. In this study, a maximum of 100% increase has been recorded. This is because fibers resist the initiation of cracks, and once they are initiated, fibers bridge them and resist their further propagation, improving the flexural capacity.
6. High-strength SCC exhibits strain softening behavior when 0.5% v/f of macro PP fibers are added to it, but this behavior changes to strain hardening, showing a further increase in load carrying capacity after the cracks are initiated when the fiber content increases to 1% and 1.5%. The behavior of concrete changes from brittle to ductile with PP fiber incorporation and shows a remarkable increase in flexural toughness. This may be because of the fibers' action, especially when the cracks are initiated, and the bridging effect of fibers comes into play, resisting the tensile stresses.

## Data Availability

The datasets used and/or analysed during the current study are available from the corresponding author on reasonable request.
